# Applications of Magnetite Nanoparticles in Cancer Immunotherapies: Present Hallmarks and Future Perspectives

**DOI:** 10.3389/fimmu.2021.701485

**Published:** 2021-10-04

**Authors:** Qingle Song, Amaneh Javid, Guofang Zhang, Yang Li

**Affiliations:** ^1^ Laboratory of Immunology and Nanomedicine, Shenzhen Institute of Advanced Technology, Chinese Academy of Sciences, Shenzhen, China; ^2^ Department of Biological Sciences, Faculty of Applied Science and Engineering, Science and Arts University, Yazd, Iran

**Keywords:** magnetite nanoparticles, macrophages, cytotoxic T lymphocytes, immune checkpoint blockade, cancer immunotherapy

## Abstract

Current immuno-oncotherapeutic protocols that inhibit tumor immune evasion have demonstrated great clinical success. However, the therapeutic response is limited only to a percentage of patients, and the immune-related adverse events can compromise the therapeutic benefits. Therefore, improving cancer immunotherapeutic approaches that pursue high tumor suppression efficiency and low side effects turn out to be a clinical priority. Novel magnetite nanoparticles (MNPs) exhibit great potential for therapeutic and imaging applications by utilizing their properties of superparamagnetism, good biocompatibility, as well as the easy synthesis and modulation/functionalization. In particular, the MNPs can exert magnetic hyperthermia to induce immunogenic cell death of tumor cells for effective antigen release and presentation, and meanwhile polarize tumor-associated macrophages (TAMs) to M1 phenotype for improved tumor killing capability, thus enhancing the anti-tumor immune effects. Furthermore, immune checkpoint antibodies, immune-stimulating agents, or tumor-targeting agents can be decorated on MNPs, thereby improving their selectivity for the tumor or immune cells by the unique magnetic navigation capability of MNPs to promote the tumor killing immune therapeutics with fewer side effects. This mini-review summarizes the recent progress in MNP-based immuno-oncotherapies, including activation of macrophage, promotion of cytotoxic T lymphocyte (CTL) infiltration within tumors and modulation of immune checkpoint blockade, thus further supporting the applications of MNPs in clinical therapeutic protocols.

## Introduction

Immuno-oncotherapy aims to activate the patient’s immune system to recognize and kill tumor cells (1). Immunotherapeutic approaches date back to ancient Egypt, with the observation that tumors could be cured with bacterial infections, an event that we now attribute to immune activation. The modern immunotherapy of cancer started with Willian B. Coley at the end of the 19th century with his “Coley’s toxin”, a mixture of killed bacteria, which non-specifically activated the immune responses, thereby resulting in tumor regression. Coley’s toxin is still used in clinical trials, and pharmaceutical companies are interested in developing modern versions of it (2, 3). Current immunotherapies include several approaches for the non-specific stimulation of innate immune mechanisms on the same line as Coley’s toxin (such as the use of BCG in bladder cancer) (4), and strategies aiming at triggering specific anti-tumor immunity. These include the development of cancer vaccines, adoptive cell therapy, and checkpoint inhibitors (5). Indeed, immuno-oncotherapy based on immune checkpoint inhibitors has revolutionized the therapy of advanced and metastatic tumors, leading to an unprecedented rate of cure (6). However, all these therapies need further development and optimization. For instance, non-specific immunological stimulation is often associated with substantial inflammation (the Coley’s toxin therapy was dubbed the “fever therapy”), and the therapy with checkpoint inhibitors that releases the block of immunity leads to uncontrolled reactions that can substantially affect the patient’s own organism and causes the unwanted immune-related adverse events (irAEs) (7). In addition, in particular for immuno-oncotherapies aiming at activating specific anti-tumor immunity, the therapeutic approach is effective only for a limited number of patients, which depended on the degree of the immunogenicity for various tumor types (8). In an effort to improve the efficacy and safety of the immuno-oncotherapy, many studies have focused on the use of engineered nanoparticles (NPs) (9–12). In this context, magnetite nanoparticles (MNPs) are already in use for medical purposes, e.g., in iron replacement therapy and magnetic resonance imaging (MRI), approved by the US Food and Drug Administration (FDA) (13, 14). The European Medicines Agency (EMA) has also approved the use of iron oxide NPs (NanoTherm^®^) for the treatment of intermittent glioblastoma multiforme (15). The suitability of MNPs as drug delivery system is based on a number of promising properties, which include their good superparamagnetism and biocompatibility, the easy synthesis and surface modulation/functionalization, and the magnetic hyperthermia and navigation capability (16–18). Based on these characteristics, MNPs can be also applied for theranostics and have been studied for a wide number of new medical applications in oncotherapy. Thus, this brief mini-review tries to report the recent promising applications of MNPs in cancer immunotherapies.

## MNPs in Tumor Imaging

Currently, FDA has approved various MNPs for clinical imaging, including Ferumoxide (19), Ferumoxtran-10 (20, 21), Ferumoxsil (22), Ferucarbotran (23), Ferumoxytol (24, 25), and Magtrace (26). However, some of these MNPs have a low performance–price ratio, and some have been withdrawn from the market because of poor clinical results. Therefore, an increasing number of studies have investigated the optimized MNPs to improve the imaging efficiency. Bai et al. (27) reported that the modification with a tumor-targeting peptide (cRGD, a molecule targeting the integrin αvβ3 overexpressed by endothelium cells of angiogenic tumor vessels) on MNPs could facilitate the effective accumulation of MNPs into the tumor of mice, thus improving the efficiency on MRI application. Moreover, MNPs can be conjugated with other imaging components to realize multi-modal imaging (28). Li et al. (29) incorporated superparamagnetic iron oxide (SPIO) NPs into 1,2-distearoyl-sn-glycero-3-phosphoethanolamine-N-[amino(poly-ethylene glycol)-5000] (DSPE-PEG5k) nanomicelles, which were further conjugated with a near-infrared fluorescence dye (Cy5) and the tumor-targeting peptide bombesin (Bom, used to target the overexpressed G protein-coupled receptors in various malignancies). These nanomicelles could target mouse MDA-MB-231 breast cancer and perform both MRI by the inner SPIO as well as the near-infrared fluorescence imaging by the Cy5 dye (29). These promising outcomes of MNPs in tumor imaging strategies demonstrated their advantages of easy surface modification and functionalization, and these properties could be further integrated into immuno-oncotherapeutic strategies. Recent studies of exploiting MNPs to enhance the efficacy of immunotherapy will be highlighted and discussed in the following paragraphs (30).

## MNPs for Enhancing Macrophage Anti-Tumor Activity

Macrophages are the very important component of the innate immune system and first-line defense cells against pathogens and cancers (31). Macrophages are both surveillance and scavenging cells that patrol the tissue and recognize/eliminate senescent, anomalous, and dead cells, and activate effector cells to kill, ingest, and degrade pathogens and tumor cells after having initiated an inflammatory process. The activities of macrophages are mainly influenced by the developmental origin, tissue of residence, and acute microenvironmental cues (32). Although the capacity as antigen-presenting cells (APCs) is weaker than the dendritic cells (DCs), tumor-associated macrophages (TAMs) could re-activate primed T cells by cross-presenting tumor antigens in the tumor microenvironment (TME) rather than in the draining lymph node, thereby contributing to the adaptive immunity in addition to their main role in innate immune responses (33). Besides, the tumor-killing functionality of TAMs should also be ideally achieved through various mechanisms, e.g., the phagocytosis of tumor cells, secretion of cytokines (e.g., IFNγ and TNFα), production of inducible nitric oxide synthase, and induction of anti-tumor inflammation (33, 34). Unfortunately, phagocytosis is partially deactivated owing to the developed escaping mechanism of overexpressing “do-not-eat-me” signal on tumors (35). Furthermore, the most representative TAMs are pro-tumorigenic M2-like phenotype cells, which suppress the anti-tumor capacity of the M1-like phenotype cells, and contribute to the tumor growth, tumor immune evasion, and metastasis (36). Therefore, a bunch of investigations that aim to promote the phagocytosis and the M1 polarization of the TAMs by the application of MNPs have been undertaken.

**Figure 1:**
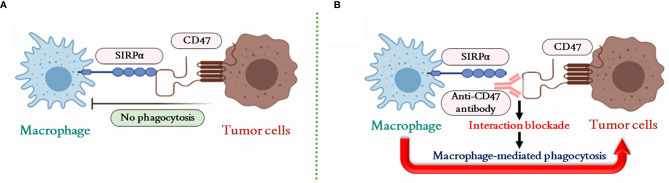
Macrophage-mediated phagocytosis regulated by the CD47–SIRPα pathway. **(A)** The CD47–SIRPα interaction inhibits macrophage-mediated phagocytosis on tumor cells. **(B)** The interaction of CD47–SIRPα can be blocked by the anti-CD47 antibody to restore the macrophage-mediated phagocytosis against tumor cells. SIRPα, signal-regulatory protein alpha; CD47, cluster of differentiation 47.

The expression of the cell surface integrin-associated protein CD47 (ubiquitously expressed on normal cells) is abundantly expressed on most tumor cells and creates a “do-not-eat-me” signal through binding with the signal-regulatory protein alpha (SIRPα) on macrophages (37–39). SIRPα is a regulatory membrane glycoprotein expressed on the macrophage membrane that accumulates at a phagocytic synapse between macrophages and tumor cells upon CD47 binding (37, 40). Thus, the blockade of the CD47–SIRPα interaction by anti-CD47 antibody could restore the macrophage-mediated phagocytosis against tumor (**Figure 1**) (41–43). Accordingly, a combined immunotherapeutic approach was proposed by using indocyanine green and sepantronium bromide co-loaded mesoporous silica NPs for photothermal and chemotherapy with primary tumors. This approach induced a primary tumor destruction that facilitated tumor antigen release and presentation for specific cytotoxic T lymphocyte (CTL) generation (44). Furthermore, MNPs coated by a silica layer conjugated with anti-CD47 antibody were subsequently magnetically guided to be accumulated at metastatic tumor sites to block the interaction of tumor-expressed CD47 with macrophages. As a consequence, macrophages recognized and destroyed/phagocytosed metastatic tumor cells in synergy with activated CTLs (44). In addition, the Fc portion of some CD47 antibody could also drive macrophage for the antibody-dependent cellular phagocytosis (ADCP), which could further strengthen the macrophage-mediated anti-tumor activity (45). However, since CD47 is an essential molecule for protecting normal cells from macrophage attack, strategies for CD47 inhibition need to be very precisely targeted to tumor cells in order to avoid severe collateral damage, as observed with anti-CD47 antibody infusion that causes thrombocytopenia or anemia (35, 45, 46).

The use of plasma membrane-coated biomimetic NPs was applied for a precise inhibition of tumor CD47 binding to macrophages (47, 48). The MNPs could be coated with membrane from genetically engineered cells that overexpressed a SIRPα variant for CD47 binding and showed a 50,000-fold increased binding affinity. These biomimetic MNPs efficiently accumulated in the TME under external magnetic field guidance and specifically blocked the macrophage-inhibiting CD47–SIRPα binding between tumor cells and macrophages (49). Such magnetic navigation approach with MNPs decreases the risk of inducing severe side effects through enhanced tumor targeting. In addition, it is known that the TME can bias the infiltrating TAMs towards a tumor-promoting M2-like phenotype (50), thereby inhibiting the cytocidal and antigen-presenting capacity of M1 macrophages (51). There are evidences that MNPs could promote the re-polarization of TAMs towards an M1 functional phenotype, which resulted in the activation of the macrophage for tumor killing (49, 52). Ferumoxytol, one of the FDA-approved MNPs, was found able to upregulate the expression of M1-related genes (*CD86* and *TNFA*) and to decrease expression of M2-related genes (*CD206* and *IL10*) in a murine macrophage-like leukemia cell line *in vitro* (52). *In vivo*, experimental tumors were restricted in their growth by treatment with ferumoxytol and showed an increased number of M1-like infiltrating macrophages in comparison to the untreated tumors (52). Other studies also exploited MNPs for inducing M1 polarization of TAMs, as recently shown with polymeric NPs coated with a membrane of LPS treated macrophage, and encapsulating Fe_3_O_4_ NPs and the imiquimod (Toll-like receptor 7 agonist, a strong macrophage activator), which were able to polarize TAMs towards M1 and achieve a concomitant restriction of experimental tumor growth (53). The membrane was prepared by the LPS stimulated macrophages to specifically target TAMs without direct M1 polarization effects that can be verified from the macrophage polarization results of PLGA-ION (PI) and membrane-coated PLGA-ION (PI@M) NPs. The CD80 expression in PI was 67.55% vs. 74.31% in PI@M. The higher M1 polarization of PI@M may be attributed to the enhanced internalization caused by the coated membrane camouflage and targeting capacity. Furthermore, vitamin C (Vc) was applied to eliminate the polarization capacity induced from the ROS pathway of enhancing p300/CBP acetyltransferase activity and promoting p53 acetylation by iron overload (54). Despite the fact that the ROS level of Vc+PI@M was reduced to 0.69-fold compared to the sole PI@M treatment, CD80 expression in PI@M (74.31%) and Vc+PI@M (73.24%) was still in a similar range, indicating that the M1 polarization was mainly caused by MNPs rather than the ROS by-product. Besides, a recent study indicated that, after taking a large amount of iron, macrophages could be polarized to M1 type by inhibiting the ERK phosphorylation (55). However, this mechanism still needs to be further investigated and confirmed critically.

Thus, MNPs have proven useful for enhancing the macrophage anti-tumor activity, both in inducing the M1 re-polarization of TAMs and as carriers of inhibitors on tumor CD47 “do-not-eat-me” signal. Although these preliminary results are promising, future research will have to overcome several issues before successfully using MNPs as macrophage activators in cancer immunotherapy. These encompass the heterogeneity of MNP effects on macrophages (56) [may show no activation effects on human primary cells (57)], the need for very accurate tumor targeting to avoid the risk of severe off-target autoimmune and self-destructive effects (which is also the major current problem of immunotherapy with checkpoint inhibitors), the need to reach distant metastatic tumors, and the assessment of efficacy in non-solid tumors.

## MNPs for Turning “Cold” Tumor to “Hot”

**Figure 2:**
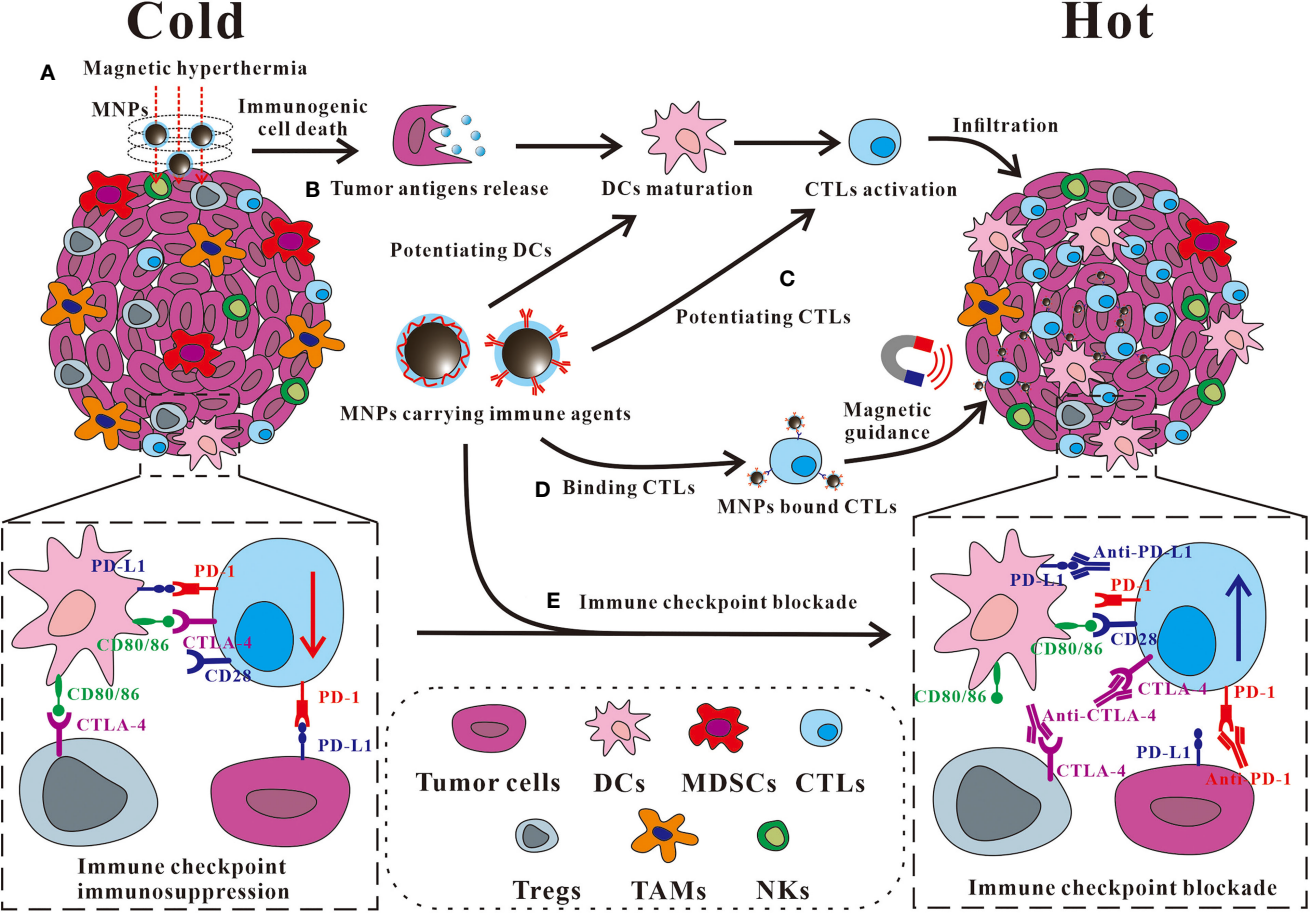
Some representative approaches of MNPs to turn a “cold” tumor into “hot”. **(A)** MNP-based magnetic hyperthermia ablation therapy is capable of inducing tumor immunogenic cell death to promote the release of antigen for DC maturation, and the subsequent CTL activation and infiltration in the tumor. **(B, C)** MNPs carrying immune agents such as agonists can be applied **(B)** for potentiating DCs or **(C)** for potentiating CTLs. **(D)** MNPs carrying immune agents such as antibodies can bind CTLs, and direct them to the tumor with an improved accumulation and infiltration by the magnetic guidance. **(E)** The tumor accumulation of the immune checkpoint antibody can be enhanced by the conjugation on MNPs for an improved immunosuppressive pathway modulation with fewer adverse effects. DCs, dendritic cells; MDSCs, myeloid-derived suppressor cells; CTLs, cytotoxic T lymphocytes; Tregs, regulatory T cells; TAM, tumor-associated macrophages; NKs, natural killer cells; PD-1, programmed cell death protein 1; PD-L1, programmed cell death ligand 1; CTLA-4, cytotoxic T lymphocyte-associated protein 4.

Besides macrophages and the innate immune cells, stimulating CTLs could be considered as another effective approach for improving the anti-tumor immune responses and was also widely studied (58). A solid tumor can be defined as “cold” or “hot”, depending on the degree and localization of tumor-infiltrating CTLs and the immunological condition of the TME (59, 60). The “cold” tumor exhibits an immune suppressive TME with an inadequate CTL infiltration and the excessively activated immunosuppressive cells, including regulatory T cells (Tregs), myeloid-derived suppressor cells (MDSCs), and TAMs (61). Meanwhile, the exhaustion of infiltrated CTLs can be induced by the immune suppressive checkpoints in TME, e.g., the programmed cell death protein 1 (PD-1)/programmed cell death ligand 1 (PD-L1) or the cytotoxic T lymphocyte antigen-4 (CTLA-4), which hindered the development of anti-tumor specific CTLs responses (62). Conversely, a “hot” tumor exhibits a high accumulation and infiltration of non-exhausted CTLs, DCs, and natural killer cells (NKs), thus being an ideal target for immuno-oncotherapy (58, 63). Thus, to improve the overall response rate (ORR) of the current immuno-oncotherapy, a growing number of MNP-based immunotherapeutic strategies aim at turning the “cold” tumors to “hot” by enhancing the number of infiltrating CTLs and modulation of immune checkpoint blockade (ICB) in TME (**Figure 2**).

### MNPs Improving CTL Tumor Infiltration

In the presence of an alternating magnetic field, MNPs can generate a magnetic hyperthermia by converting the magnetic energy into thermal energy, which can provoke immunogenic/inflammatory cell death in the tumor (64, 65). The hyperthermia may induce immunogenic cell death (ICD) to release tumor-associated neoantigens and danger-associated molecular patterns (DAMPs), including the exposure of calreticulin (CRT), or the release of chromatin-binding protein high mobility group B1 (HMGB1) and the adenosine triphosphate (ATP) (66). Subsequently, the recognition and processing of the neoantigens can be promoted and followed with an effective T-cell priming (67). Thus, after the primary tumor elimination by the magnetic hyperthermia of the ferromagnetic iron oxide nanorings, the “eat-me” signal of CRT was elicited on the surface of the immunogenic dying 4T1 tumor cells and promoted the macrophage activation, which further enhanced the activation and infiltration of CTLs in the re-challenged or metastatic tumor on mice (68). Besides, as the most powerful APCs, the activation of DCs is crucial for promoting the infiltration of CTLs. To achieve a more effective tumor antigen uptaken by DCs, an MNP-based vaccine has been developed using Fe_3_O_4_ nanocluster core loading with the CpG oligodeoxynucleotide (CpG-ODN, a Toll-like receptor 9 agonist) and tumor cell membrane shell decorated with anti-CD205 for preferentially DC recognition (69). These DC targeting MNPs could extend the retention in lymph nodes by the magnetic control for an increased DC internalization. The CpG-ODN and the various antigens on the tumor cell membrane paved the way of DC maturation, thus facilitating the MHC cross-presentation and T-cell activation, eliciting a tremendous amount of CTL infiltration and responses in the tumor (69). Besides promoting the activation of DCs and CTLs, the possibility of binding MNPs on the surface of CTLs to improve the tumor infiltration by a magnetic field near the tumor site was also explored. By coating Fe_3_O_4_ nanoclusters with the leucocyte membranes modified with the co-stimulatory ligand CD28 antibody (αCD28) and peptide (SIINFEKL)-loaded major histocompatibility complex class-I (pMHC-I), the artificial antigen-presenting cells (aAPC) were constructed that successfully targeted CTLs for stimulation (70). Then, the CTLs that bound these nanoclusters could be effectively directed to tumor with an increased accumulation and infiltration facilitated upon magnetic guidance, resulting in an enhanced tumor killing efficiency (70).

### MNPs Modulating Immune Checkpoint Blockade

Based on the current progress in immuno-oncotherapy, ICB therapy, e.g., PD-1/PD-L1 and CTLA-4, has exerted a major therapeutic effect on patients with advanced cancers (71). Immune checkpoints exhibit crucial functions in adjusting the balance of immune homeostasis and preventing T-cell-mediated autoimmune diseases, while they are also found hijacked by the tumor for suppressing the anti-tumor immune responses (72, 73). The PD-1 on T cells can be activated by binding its ligand PD-L1 on tumors, resulting an exhausted condition of infiltrated CTLs. CTLA-4, a new immunoglobulin superfamily candidate on T cells (including CD4, CD8, and Treg cells), restrains the co-stimulatory signals of CD28-CD80/86 by competitively binding with CD80/86 on antigen-presenting cells (65, 74). In clinical practice, anti-PD-1/PD-L1 and anti-CTLA-4 therapy proved effective for restoring the anti-tumor T-cell-mediated immune responses (75). However, ICB therapy only shows limited ORR with commonly associated irAEs, which limited its clinical application (76). Therefore, the combinational immuno-oncotherapy based on the multi-functional MNPs attracts considerable attention because of the immuno-stimulation effects of magnetic hyperthermia and the drug delivery capability of immuno-stimulatory adjuvant or checkpoint antibodies for the improved activation of CTLs and the accumulation of ICB antibodies (77).

The magnetic hyperthermia induced by MNPs could cause tumor ablation and the release of neoantigens. With the help of imiquimod (a toll-like receptor 7 agonist), CTLs were then effectively activated (78, 79). Thus, a further combinational approach by applying immune checkpoint antibodies, such as anti-CTLA-4 for Treg suppression or anti-PD-1/PD-L1 to restore exhausted CTLs, could be used for the synergistic oncotherapy to eliminate primary tumor and inhibit tumor metastasis, or even to generate a robust immunological memory for tumor recurrence prevention without noticeable systemic toxicity (78, 80). Beside the combination with checkpoint inhibitors separately, conjugating checkpoint inhibitors on MNPs demonstrated a better tumor targeting and treatment efficiency. The checkpoint inhibitor (anti-PD-L1) and CTL activators (anti-CD3 and anti-CD28) could be conjugated on the fucoidan-dextran-coated iron oxide NPs for an improved tumor accumulation by the magnetic guidance (81). These MNPs mediated ICB therapy-enhanced PD-L1 blockade and promoted CTL activation that prolonged the median survival time of tumor-bearing mice from 32 to 63 days with minimized adverse events owing to a much lower anti-PD-L1 dosage used than the soluble anti-PD-L1 treatment (81). Remarkably, the superparamagnetic iron nanoclusters were armed with PD-1 antibody by TME pH-sensitive bond and successfully bound on CTLs *in vitro*. These MNP/CTL cells could be magnetically guided to the solid tumors in mice for improved CTL infiltration and tumor killing, and further aided with PD-1 antibody release through acid-mediated MNPs, for a synergistically enhanced immuno-oncotherapy with minimal side effects (82).

As these studies indicated, magnetic hyperthermia therapy induced by functionalized MNPs can effectively promote the immunological stimulation, tumor infiltration of CTLs, and the efficiency of ICB therapy. Moreover, various stimuli or ICB antibodies can be conjugated with the MNPs for improved targeting and immune cell activation in solid tumor. Meanwhile, the targeting cells, such as CTLs, attached with MNPs carrying ICB antibody on their surface, can be magnetically directed to tumor site for improved tumor accumulation. Such strategy has been proved successfully to enhance both CTL infiltration and ICB efficiency in one shot.

## Conclusions and Future Perspectives

Taken together, extensive progresses have been made with various MNP platforms toward different applications for the enhanced immuno-oncotherapy. MNPs exhibited good capabilities for magnetic hyperthermia, magnetic navigation, and immuno-agent delivery to enhance macrophage anti-tumor capacity, to increase the tumor infiltration of CTLs, and to improve ICB efficiency. Moreover, the CTLs that adhered to the MNPs carrying immune checkpoint antibodies obtained the ability of magnetic navigation as well, which could greatly improve the CTL infiltration and ICB effects in solid tumors. These MNP-based immunotherapies exhibited higher anti-tumor efficiency with fewer side effects in the experimental studies. In the future therapeutic approaches of MNPs, the combination of these unique properties possessed by MNPs may be the most promising strategy for successful cancer therapeutics. The hyperthermia induced by MNPs can eliminate tumor cells directly, which provokes the ICD from DAMPs and antigen release for the improved anti-tumor immune responses. Moreover, the tumor tissue-retained MNPs could polarize the remaining TAMs to M1 macrophage as a further method to eliminate the residual tumor effectively. Consequently, the tumor metastasis and recurrence may be efficiently and sustainably inhibited by the synergistic strategy. Despite these advantages, disadvantages of high difficulty in mass production, lack of the precise analytic methods for examining the properties, and the complicated usage condition/equipment should be noticed for the future MNP application in oncotherapy. Moreover, in the clinical trials, only few MNP formulations, e.g., ferumoxytol, have shown new progress as an MRI-enhancing agent. Meanwhile, after the withdrawal of some MNPs in clinic, various regulations have currently been announced by the regulatory agencies, to assure a safe, effective, and quality controllable MNPs for fundamental and translational development. With this concern, immense information needs to be researched to cope with the complex challenges, e.g., nano-bio interactions in the body, crossing physiological and tumor-specific technical hurdles, stealth behavior against late endosomal/lysosomal system into the tumor cytosol, and boosting the desired immunity without severe side effects or only with short-term adverse effects. Moreover, MNPs still have to face a number of biological barriers, such as the “Mononuclear Phagocyte System (MPS)”, that abate the accumulation and localization of MNPs at the targeted tumor site, and limit their further application as efficacious immunotherapeutic vehicles. However, as promising outcomes have been seen from the current MNP synergetic immuno-oncotherapy, we believe that more potential approaches will be continuously exploited for ideal functionalized MNPs with successful clinical achievements.

## Author Contributions

YL conceived the idea of the review. QS and AJ performed the literature search, prepared the figures, and wrote the draft. GZ contributed to revising and editing the review. YL contributed to the critical revising, rewriting, and editing of the review. All authors contributed to the article and approved the submitted version.

## Funding

This work was financially supported by the Shenzhen Science and Technology Program (GJHZ20190821155803877), the National Natural Science Foundation of China (32171390 and 31701005), the China Postdoctoral Science Foundation (2020M672898), the SIAT Innovation Program for Excellent Young Researchers (E1G068), and CAS President’s International Fellowship Initiative (PIFI, 2020VBA0028, 2020VBA0022, 2022VBA0008 and 2021PB0060).

## Conflict of Interest

The authors declare that the research was conducted in the absence of any commercial or financial relationships that could be construed as a potential conflict of interest.

## Publisher’s Note

All claims expressed in this article are solely those of the authors and do not necessarily represent those of their affiliated organizations, or those of the publisher, the editors and the reviewers. Any product that may be evaluated in this article, or claim that may be made by its manufacturer, is not guaranteed or endorsed by the publisher.
